# Treatment of Gout with TCM Using Turmeric and Corn Silk: A Concise Review Article and Pharmacology Network Analysis

**DOI:** 10.1155/2022/3143733

**Published:** 2022-10-14

**Authors:** Haoyu Zhang, Huizhong Jiang, Mengya Zhao, Yan Xu, Jiabin Liang, Yufeng Ye, Hanwei Chen

**Affiliations:** ^1^Guangzhou University of Chinese Medicine, Guangzhou 510405, China; ^2^Guangzhou Panyu Central Hospital, Guangzhou 511400, China; ^3^Guizhou University of Traditional Chinese Medicine, Guiyang 550000, China; ^4^School of Pediatrics, Henan University of Chinese Medicine, Zhengzhou 450000, China; ^5^Panyu Health Management Center (Panyu Rehabilitation Hospital), Guangzhou 511495, China

## Abstract

**Objective:**

This work aimed to study the compounds, targets, and pathways of turmeric and corn silk for gout and to explore the mechanism of “the same disease with different treatments” based on network pharmacology and molecular docking.

**Methods:**

We used the TCMSP, PubChem, and SEA databases to screen the compounds and targets of turmeric and corn silk, gout-related proteins through TTD, Drugbank, DisGeNET, GeneCards, OMIM, and PharmGkb, and used Cytoscape to construct a “compound-target-disease” network. Then, we constructed a protein-protein interaction network (PPI) and used Metascape to perform GO and KEGG analysis. Finally, molecular docking (SYBYL) was used to verify the degree of binding between key targets and compounds.

**Results:**

We found bisacumol, campesterol, and stigmasterol to be the main turmeric compounds that exerted a marked effect on gout treatment by targeting protein processing in the endoplasmic reticulum through the HSPA1B, HSP90AB1, and STUB1 proteins. The main corn silk compound, Mandenol, treated gout by targeting the Hippo signaling pathway through the CTNNB1, YWHAG, and YWHAZ proteins.

**Conclusion:**

Turmeric and corn silk can treat the same disease, gout, through different pathways and targets. The scientific connotation of “same disease with different treatments” can be preliminarily clarified by analyzing targets and pathways.

## 1. Introduction

With the global outbreak of COVID-19 in 2020, lifestyles changed significantly. Success in the treatment of the disease achieved by the combined use of traditional Chinese medicine (TCM) and modern medicine garnered the attention of society for TCM. As one of the four ancient civilizations, China has a lengthy history, with a traditional health-preserving and TCM culture. TCM is a treasure house of natural herbs. In recent years, its application worldwide has increased dramatically due to its excellent therapeutic effects and a paucity of side effects. The equilibrium state is one of TCM's main theories and includes the balance of the human body, the balance between man and nature, and the balance between man and the social environment [1]. Once the balance is disturbed, the human body will produce various pathological substances, such as static blood, phlegm, and dampness, and further produce different symptoms. Therefore, various treatments are adopted according to different symptoms (syndrome differentiation), which is the basis of TCM [2]. During treatment, different methods are adopted according to various conditions, i.e., different treatments for the same disease [3]. The application of “same disease with different treatments” reflects the characteristics of TCM in the diagnosis and treatment, emphasizing syndrome differentiation.

Gout, the most common inflammatory arthritis, is caused by hyperuricemia, which causes sodium urate crystals to deposit in joints, tendons, and other tissues [4]. Several recent reports have shown that the worldwide prevalence of gout is between 1 and 6.8%, and these percentages are rising [5]. In addition, many studies have shown that gout is a risk factor for diseases such as hypertension, diabetes, cardiovascular and cerebrovascular diseases, and chronic kidney disease, and it is also a predictor of premature death [6]. Gout treatment primarily involves antiinflammatory and analgesic treatment in acute arthritis, uric acid control in the chronic phase, and daily personal life management [7]. Medications for acute gout attacks include colchicine, nonsteroidal antiinflammatory drugs (NSAIDS), steroids, and biological agents [7]. Although these are recommended as first-line drugs for the clinical treatment of gout, they are restricted due to their respective liver and kidney toxicities and gastrointestinal adverse reactions [8]. Biological agents, such as interleukin-1 blockers, can achieve better therapeutic results with minimal side effects, but are expensive [9]. Lowering uric acid requires persistence, but most of the above drugs are challenging to use for a long time due to toxic side effects. Therefore, more natural products need to be found for gout treatment.

Gout (*tong-feng*) (also known as *damp bi*) is a common disease name used in TCM and modern medicine; in TCM, both gout and hyperuricemia are in the category of gout [10]. Furthermore, in TCM, the development of gout is related to congenital liver and kidney deficiency and the accumulation of dampness, phlegm, and static blood, resulting in the formation of dampness syndrome, phlegm syndrome, and blood stasis syndrome, respectively, as well as kidney deficiency syndrome. Based on the syndrome, different methods, such as the promotion of blood circulation, diuresis, and invigorating the kidney, are adopted [11]. Through syndrome differentiation, effective Chinese medicines for gout treatment have been discovered, such as turmeric [12], corn silk [13], and tuckahoe [14]. Both clinical applications and modern pharmacological research have confirmed the uric acid-lowering effect of these herbs. Through literature research, it has been found that turmeric and corn silk are effective medicines for gout and are also part of the concept of “medicine and food homology” [15, 16]. The coexistence of nutrients and active ingredients is strong evidence for the dual use of medicine and food. They avoid the drug's toxicity and side effects and simultaneously restore or maintain human health through daily intake of the prescribed dose [16].

This study adopted the network pharmacology method, taking turmeric and corn silk as examples. Constructing a drug-component-target-disease network reveals the mechanism of “the same disease with different treatments” to explain the scientific connotation of TCM and the pharmacological mechanism of Chinese medicine (Figure S1, Supporting Information).

## 2. Materials and Methods

### 2.1. Prediction of Turmeric and Corn Silk Compounds and Their Targets

The turmeric and corn silk components were collected through the herbal platform TCMSP and screened according to absorption, distribution, metabolism, and excretion (ADME) parameters. The screening criterion was oral bioavailability (OB) ≥ 30% and drug-likeness (DL) ≥ 0.18, and this was combined with relevant literature to supplement the compounds. In addition, the PubChem database was used to confirm the molecular structure of the compounds, and TCMSP targets and the SEA database prediction model were used to predict the possible targets of the compounds (Table 1).

### 2.2. Collection of Disease Targets

The keywords “hyperuricemia” and “gout” were used, and “Homo sapiens” was selected for the species. Next, the keyword search was conducted in the Drugbank, DisGeNET, GeneCards, OMIM, and PharmGkb databases. Lastly, duplicate genes were deleted.

### 2.3. Network Construction

Cytoscape [17] was used to construct the “medicine-compound-target-disease” network of turmeric and corn silk, where “node” was used to represent the compounds or target, and “edge” was used to represent the relationship. The NetworkAnalyzer (Cytoscape plug-in) was used to analyze the network characteristics, including the degree, betweenness, and closeness, to study the relationship between the compounds and targets.

### 2.4. Protein-Protein Interaction (PPI) and Candidate Target Screening

The Cytoscape plug-in BisoGenet was used to construct a protein-protein interaction (PPI) network for compound potential and disease targets. Merge was used in the software to fuse the two network diagrams and extract the intersection. The direct and indirect intervention target regulation network graphs for turmeric and corn silk were obtained for gout. The plug-in CytoNCA [18] in Cytoscape was used to screen the degree, betweenness, closeness, LAC, and network, with the degree ≥2 times the median as the condition, and the PPI node was selected. With the degree, betweenness, closeness, LAC, and network greater than or equal to the median as the condition, the turmeric, and corn silk candidate targets for gout were selected.

### 2.5. Module Analysis Using GO and KEGG

Metascape [18] is a tool that integrates multiple databases such as Gene Ontology (GO), Kyoto Encyclopedia of Genes and Genomes (KEGG), and Drugbank, which can be used for biological process annotation and pathway analysis and can mainly guarantee the timeliness of the data. The above-mentioned candidate targets were imported into Metascape, and the species was set to *H. sapiens* for analysis. The results of protein interaction and module analysis, GO enrichment analysis, and KEGG pathway analysis were retained, and the results were sorted according to the number of targets. Finally, the top results were retained and analyzed, and *R* package [19] visualization was performed.

### 2.6. Molecular Docking

SYBYL-X [20] was used to optimize proteins and small molecules according to the related molecular docking literature [21], for protein processing, while the Surflex-Dock module was used for molecular docking. The compound and target protein interaction was scored according to the Total-Score scoring function. The larger the Total-Score value, the better the matching and binding effect of the compound and the protein. With a Total-Score >5 as the threshold, Pymol was used to select the best results of the two medicines' protein binding for graphing.

## 3. Results

### 3.1. Turmeric and Corn Silk Compounds and Targets

The three turmeric and twelve corn silk compounds were screened through the TCMSP database and supplemented by turmeronol A, turmeronol B, and bisacumol in turmeric [22]. Their OB and DL values were less than the screening conditions and were deleted by the system, but they were included in the search literature, given their potential significance. A total of six turmeric compounds were included in the follow-up study. The potential targets of the compounds were queried through the TCMSP and SEA databases, and after deleting duplicates, 31 turmeric targets and 92 corn silk targets were obtained. As shown in Table 2, the potential turmeric compounds were stigmasterol, campesterol, and bisacumol, among others.  Table 3 shows that the potential compounds of corn silk include mandenol, schottenols, and luteolin.

### 3.2. Disease Targets of Gout

The keywords “hyperuricemia” and “gout” were entered into the TTD, Drugbank, DisGeNET, Genecards, OMIM, and PharmGkb databases to obtain gout-related targets. The numbers of disease-related targets were 20, 66, 258, 2319, 13, and 4, respectively, and after deduplication were 20, 38, 234, 1971, 13, and 4, respectively. A total of 2081 targets were obtained. After using *R* studio 4.0.5 to cross the compound targets of turmeric and corn silk with the disease targets of gout, we get 6 and 36 intersection targets, respectively. The above targets are presented as a Venn diagram (Figure S2, Supporting Information) with the *R* packages [19].

### 3.3. “Medicine-Compounds-Target-Disease” Network

We import the turmeric and corn silk compounds and target gene symbols into Cytoscape 3.8.2 to obtain the network diagrams of turmeric and corn silk for gout, involving 15 nodes and 34 edges (turmeric), 49 nodes, and 62 edges (corn silk), respectively. In the nodes, purple represents disease, yellow represents turmeric (JH), corn silk (YMX), green represents compounds, and red represents the targets of turmeric and corn silk for gout (Figure S3, Supporting Information).

### 3.4. Construction of PPI Networks

BisoGenet constructed the PPI network of turmeric and corn silk for gout. The results showed that: turmeric candidate targets can interact directly or indirectly with 1,101 targets, and their correlations can reach 17095; corn silk candidate targets can interact with 5145 targets, have direct or indirect effects, and their correlations can reach 109,046; there were 13010 targets directly or indirectly related to gout, and there were 241,154 interconnected targets. The intersection of the disease and targets was shown in Figures S4 and S5 (Supporting Information). After calculating the attribute value for the network topology characteristic of the intersection PPI, turmeric and corn silk targets were screened twice, and 32 and 10 candidate targets were obtained, respectively. As shown in Table 4, candidate targets of turmeric for gout were HSPA8, VCP, HSP90AB1, HSPA5, HSPA1B, NFKB1, and STUB1. As shown in Table 5, it could be seen that the candidate targets of corn silk for gout were YWHAZ, CTNNB1, YWHAG, and NPM1.

### 3.5. Module Description of Turmeric and Corn Silk for Gout

**Figure 1:**
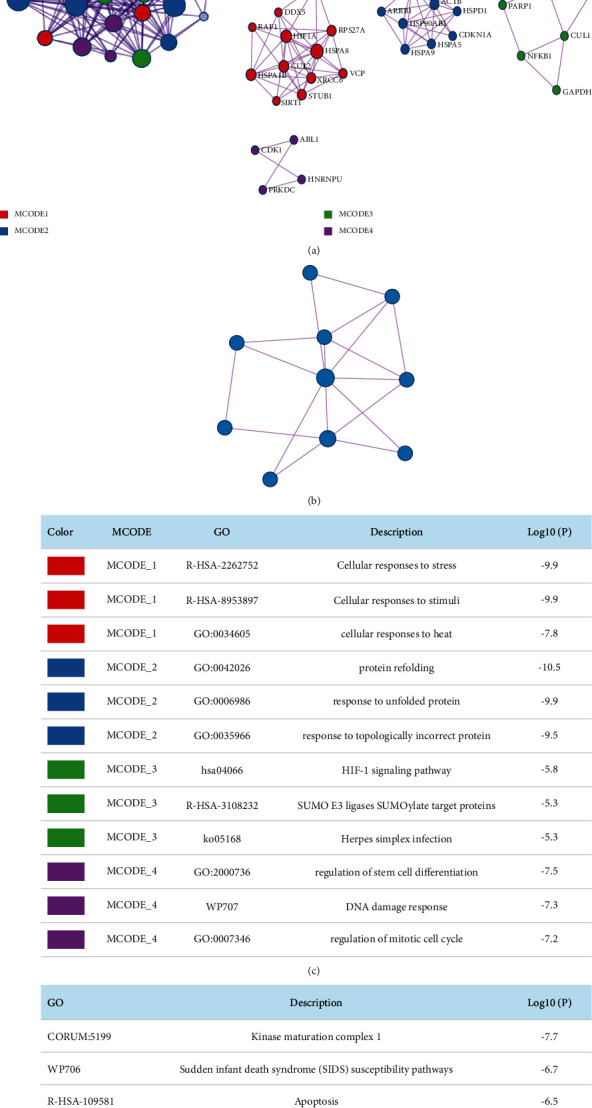
Module analysis results. (a) Interaction network and module analysis of target proteins of turmeric for gout. (b) Interaction network and module analysis of target proteins of corn silk for gout. (c) Module network function description of turmeric for gout. (d) Module network function description of corn silk for gout.

The candidate targets obtained mentioned above were imported into the Metascape database, the interaction relationship was analyzed through the molecular complex detection algorithm, and the module was obtained. In the module analysis results, turmeric involves 4 modules, 32 nodes, and 240 edges; corn silk involves 1 module, 10 nodes, and 18 edges. A functional description of the biological process in the module suggests that these targets may play an essential role in the treatment of gout through turmeric and corn silk. The results are shown in Figure 1.

### 3.6. GO Enrichment Results and KEGG Pathway Enrichment Results

R packages (“ggplot2” and “clusterProfiler”) [19] were used to perform gene enrichment analysis on the above 32 (turmeric) and 10 (corn silk) candidate targets, including GO's biological process (BP) and cellular component (CC), molecular function (MF), and KEGG pathways. Bar and bubble graphs were compiled for the BP, CC, and MF results, and *R* package was used to draw column charts and bubble plots for the KEGG pathway results. The bubble color from red to blue reflected the *p* value from small to large. The size of the bubble indicated the number of genes in this pathway, and the numbers on the bottom were the proportions of genes. Simultaneously, visual analysis of candidate targets and the primary GO function and KEGG pathway enrichment were performed, and the principal enrichment pathway diagrams were presented.

**Figure 2:**
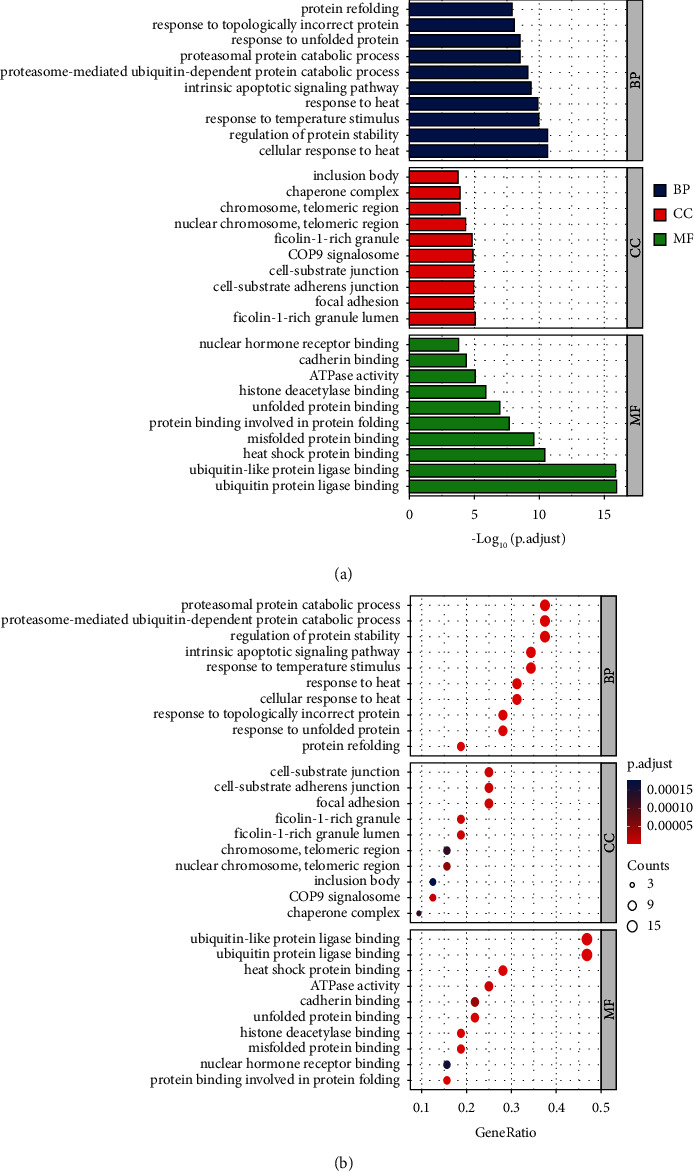
GO functional enrichment analysis of turmeric targets. (a) Column chart. (b) Bubble plot.

**Figure 3:**
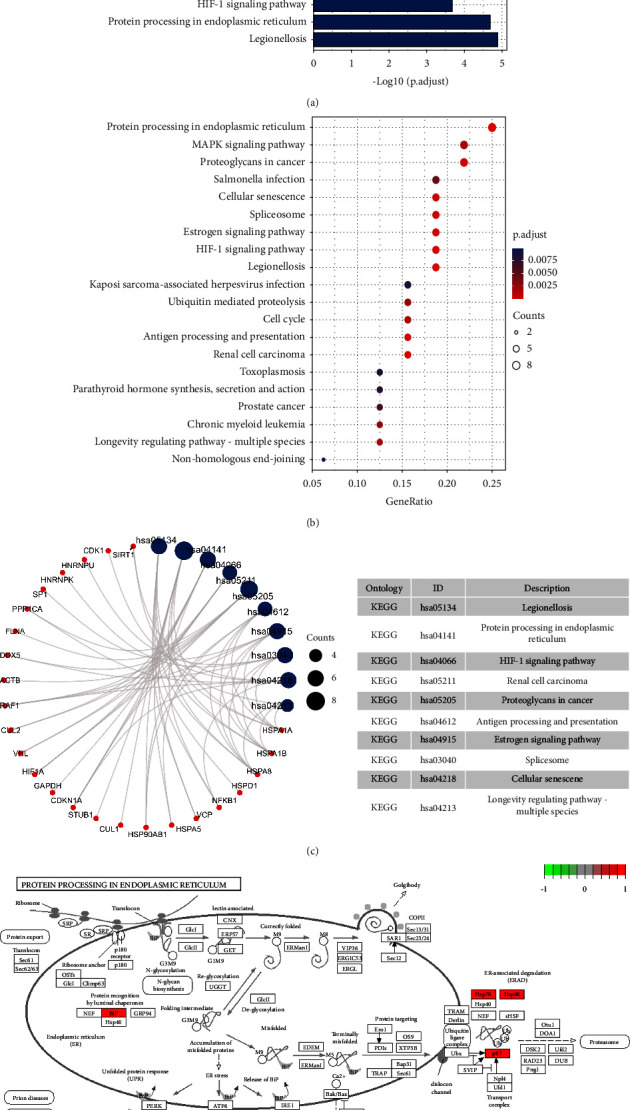
KEGG pathway enrichment analysis of turmeric targets. (a) Column chart. (b) Bubble plot. (c) KEGG pathway enrichment analysis results. (d) KEGG pathway diagram.

As shown in Figure 2, the biological processes involved in turmeric's 32 targets are enriched in the cellular response to heat and the regulation of protein stability. The genes are located in the inclusion body, ficolin-1-rich granule, and their molecular functions include ubiquitin protein ligase binding, heat shock protein binding, and unfolded protein binding.  Figure 3 shows the KEGG pathway enrichment analysis of turmeric. Twenty results were selected based on their *p* values, and these were primarily involved in protein processing in the endoplasmic reticulum, the HIF-1 signaling pathway, and the MAPK signaling pathway.

**Figure 4:**
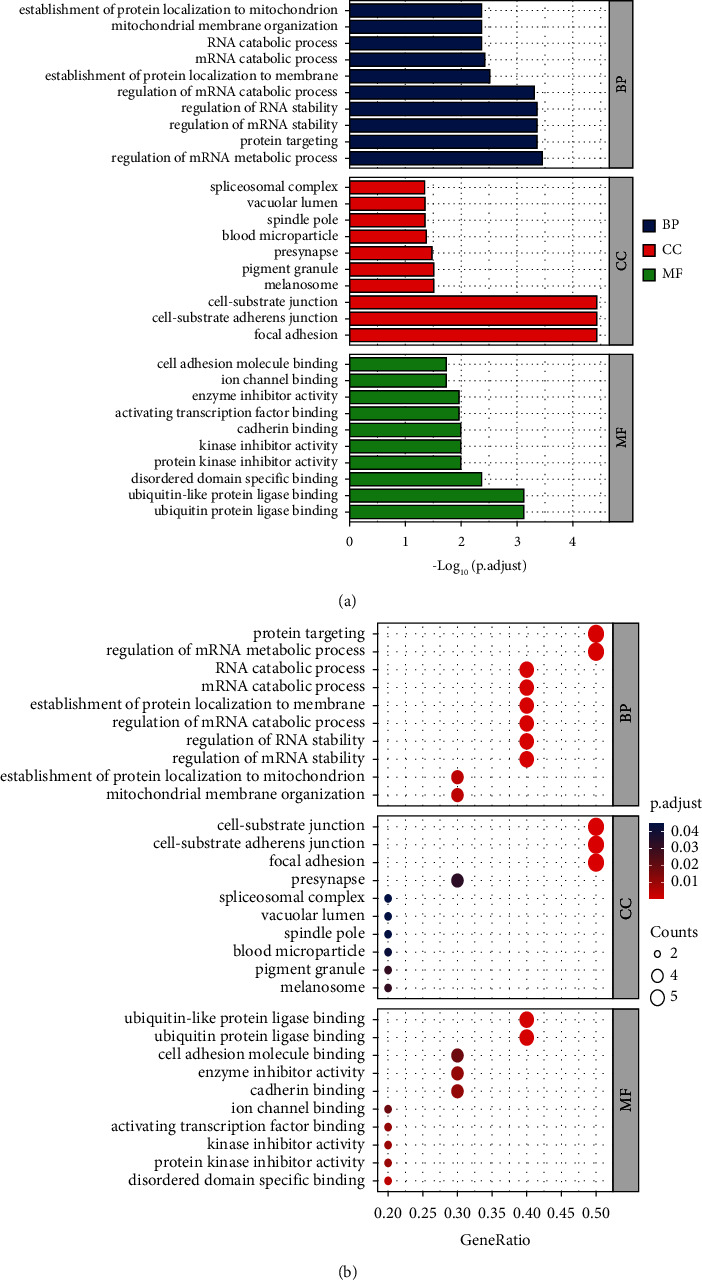
GO functional enrichment analysis of corn silk targets. (a) Column chart. (b) Bubble plot.

**Figure 5:**
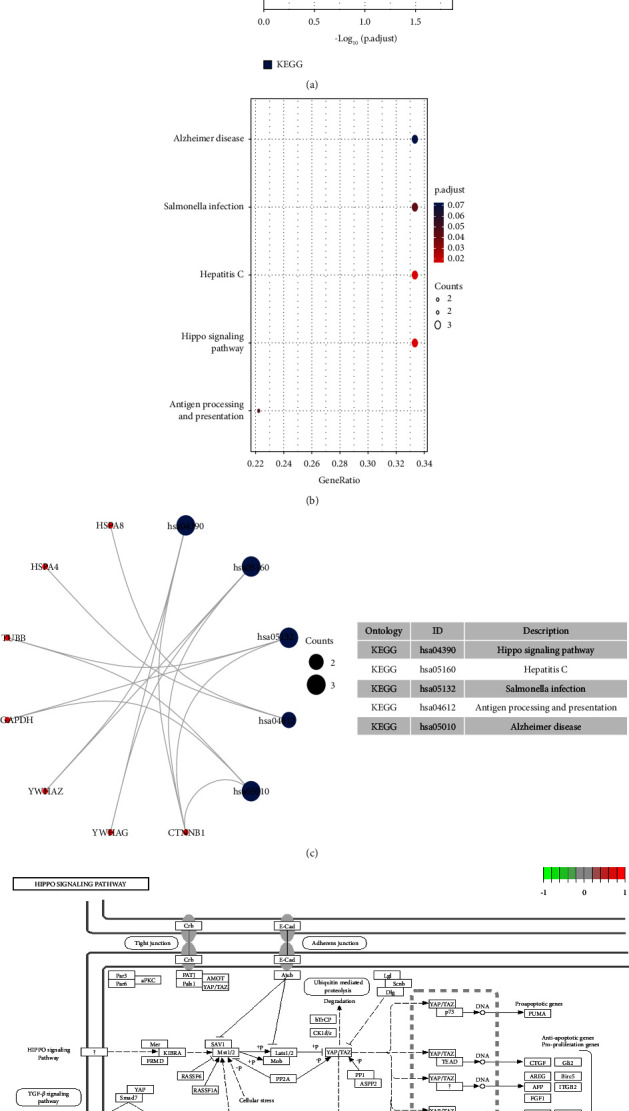
KEGG pathway enrichment analysis of corn silk targets. (a) Column chart. (b) Bubble plot. (c) KEGG pathway enrichment analysis results. (d) KEGG pathway diagram.

As shown in Figure 4, the biological processes involved in the ten corn silk targets are enriched in protein targeting, mRNA, and RNA biological processes, with genes coding for focal adhesion, cell-substrate junction, and their molecular functions, including ubiquitin protein ligase binding and protein kinase inhibitor activity.  Figure 5 shows the KEGG pathway enrichment analysis for corn silk. Five results were selected based on their *p* values. These compounds are primarily involved in the Hippo signaling pathway, antigen processing and presentation, and the cell cycle.

### 3.7. Molecular Docking

The results of the KEGG analysis showed that potential turmeric compounds might play a role in the treatment of gout by targeting protein processing in the endoplasmic reticulum signaling pathway, and potential corn silk compounds may play a therapeutic role by targeting the Hippo signaling pathway. We used SYBYL-X2.0 software [20] to verify these results and dock all targets enriched in protein processing in the endoplasmic reticulum pathway with the turmeric compounds. In addition, all targets enriched in the Hippo signaling pathway and potential corn silk compounds were docked. The results are shown in Table 6. A T_score >7 indicates that the ligand molecule binds to the receptor protein with high activity, and a T_score ≥5 indicates that it has good binding activity.

**Figure 6:**
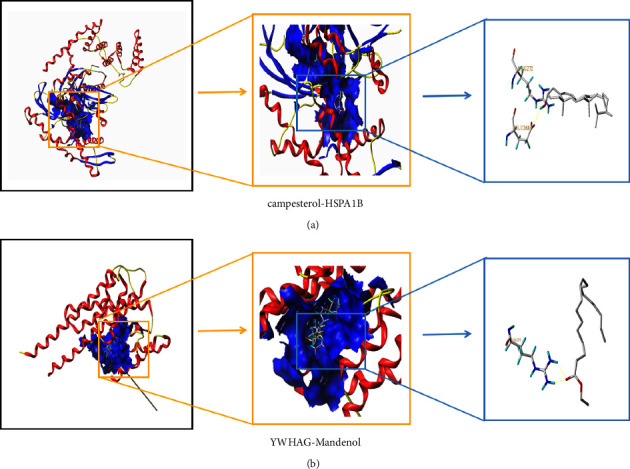
Docking diagram. (a) Turmeric. (b) Corn silk.

The scoring results showed that the turmeric, bisacumol, campesterol, and stigmasterol compounds had high activity when docking with the target HSPA1B and good activity when docking with the STUB1 and HSP90AB1 targets, suggesting that bisacumol, campesterol, and stigmasterol may be the key compounds of turmeric for gout. Mandenol, the corn silk compound, had high activity when docking with the target YWHAG and good activity when docking with the CTNNB1 and YWHAZ targets, suggesting that Mandenol may be an essential corn silk compound for gout (Table 6). For example, as shown in Figure 6(a) is the docking diagram of the turmeric target HSPA1B and the compound campesterol, and B is the docking diagram of the corn silk target YWHAG and the compound Mandenol.

## 4. Discussion

In this study, the potential mechanism of gout treatment using turmeric and corn silk was investigated using the network pharmacology method, including compound target network construction, PPI network analysis, GO enrichment analysis, and KEGG pathway analysis.

Network analysis of compound targets and molecular docking showed that bisacumol, campesterol, and stigmasterol might be the key turmeric compounds, and Mandenol might be the key corn silk compound. Bisacumol is a sesquiterpenoid [23]. The sesquiterpene compounds of turmeric have strong antimicrobial, antiinflammatory, neuroprotective, anticancer, antiviral, and antithrombotic activities [24]. Campesterol can be antiinflammatory, antiangiogenic, anticancer, antioxidant, and cholesterol-lowering [25, 26]. In addition, campesterol reduces the accumulation of proinflammatory phospholipids in the intestine and prevents the influx of mucosal myeloperoxidase-positive (MPO) cells, thereby inhibiting inflammation [26]. Stigmasterol can significantly suppress the expression of proinflammatory mediators (TNF*α*, IL-6, IL-1*β*, iNOS, and COX-2) and increase the expression of antiinflammatory cytokines (IL-10) in the joints of arthritic rats by down-regulating the expression of NF-kBp65 (inhibiting *p*-IKB-*α* activation) and p38MAPK [27]. Mandenol (ethyl linoleate), the active ingredient in corn silk, is an essential fatty acid with antibacterial and antiinflammatory properties [28]. The above compounds have antiinflammatory effects, suggesting that they may be key compounds in the treatment of gouty arthritis.

Our PPI analysis showed that turmeric and corn silk influence gout through their impact on a complex biological network, including HSPA1B, HSP90AB1, STUB1, CTNNB1, YWHAG, and YWHAZ. Furthermore, the results of molecular docking indicate that the above compounds can also be combined with turmeric and corn silk. The essential turmeric targets HSPA1B and HSP90AB1 belong to the heat shock proteins (HSP) family, which is thought to play an essential role in the immune response. Studies have shown that HSPA1B inhibits viral proliferation in a viral infection's middle and late stages [29]. HSP90AB1 is a subtype of HSP90, an intracellular chaperone and is known to regulate inflammatory processes, including the NLRP3 inflammasome and secretion of the proinflammatory cytokine interleukin (IL)‐1*β* [30]. STUB1 belongs to the ligase class, has ligase activity, and participates in regulating energy metabolism pathways and metabolism. A study showed that silencing STUB1 increased apoptosis of HK-2 cells and the proinflammatory cytokine production of IL6, TNF*α*, and IL1*β* induced by cisplatin [31]. The critical target of corn silk, CTNNB1 (*β*-catenin), is a member of the cyclic catenin family, whose primary role is to regulate adhesion between cells and intercellular substances. In addition, some studies have indicated that activation of the canonical Wnt-1/*β*-catenin pathway regulates the immune response and induces appropriate T cell responses [32]. YWHAG and YWHAZ belong to the YWHA protein family. Studies have shown that long-chain noncoding RNA NORAD has a protective effect on brain injury and inflammation induced by cerebral ischemia/reperfusion injury by regulating miR-30a-5p/YWHAG [33]. When cells are in an unfavorable living environment (e.g., hypoxia), it can regulate cell autophagy, promote DNA damage repair, inhibit cell apoptosis, and protect cells from stress damage [34]. Studies have shown that the down-regulation of YWHAZ can reduce the inflammatory response [35]. All of the above targets can regulate the immune or inflammatory response, suggesting a role for turmeric and corn silk in gout treatment.

By referring to GO and KEGG pathway enrichment analyses for turmeric and corn silk targets for gout, module and overall analyses' results were found to be the same, indicating that protein processing in the endoplasmic reticulum, HIF-1 signaling pathway, and Hippo signaling pathway is important. The endoplasmic reticulum (ER) is a subcellular organelle in which proteins are folded with the help of luminal chaperones. Accumulating misfolded proteins in the ER causes ER stress and activates a signaling pathway called the unfolded protein response (UPR). Studies have shown that tophi can promote osteoclast differentiation and proliferation by a mechanism closely related to ER stress [36]. This process shows that the protein processing in the ER is closely related to gout. In addition, research has suggested that hypoxia can cause an increase in purine metabolites (uric acid, xanthine, and hypoxic purine). Hypoxia is closely related to the hypoxia-inducible factor 1 (HIF-1) signaling pathway [37]. HIF-1 plays an essential role in executing an optimal inflammatory response by immune cells [38]. The Hippo signaling pathway is an evolutionarily conserved signaling pathway that participates in critical biological processes such as the size control and development of different organs in mammals, tissue regeneration, and stem cell regulation [39]. The Hippo pathway plays a crucial role in maintaining homeostasis, inflammation-induced regeneration, and innate immunity [40]. In summary, turmeric may have a therapeutic effect on gout through protein processing in the endoplasmic reticulum and HIF-1 signaling pathway, and corn silk may act through the Hippo signaling pathway.

Turmeric is the dried rhizome of *Curcuma longa* L., a member of the ginger family [41] recorded in the “Compendium of Materia Medica” and has the effects of promoting qi, breaking stasis, and unblocking meridians to relieve pain [42]. In addition, turmeric is widely used as a food. Its powder can be used as a spice in curries, soups, noodles, and steamed buns to enhance the fragrance, and it can also be used to make turmeric latte, turmeric milkshakes, and other beverages [43]. Shen et al. found that the curcumin group could significantly reduce the levels of uric acid (UA), xanthine oxidase (XOD), creatinine (CRE), and blood urea nitrogen (BUN) in hyperuricemic mice compared with the model group (*p* < 0.01), significantly down-regulating glutamic oxaloacetic transaminase (AST) and glutamic pyruvic transaminase (ALT) levels (*p* < 0.05), and improving liver and kidney tissue morphology (*p* < 0.05) [44]. Chen et al. found that curcumin treatment markedly inhibited the activation of the NF-*κ*B signaling pathway and expression levels of the NF-*κ*B downstream inflammatory genes such as IL-1*β*, IL-6, TNF*α*, COX-2, and PGE2 (*p* < 0.05) in MSU-stimulated THP-1-derived macrophages [45]. Furthermore, intraperitoneal administration of curcumin alleviated MSU crystal-induced paw and ankle joint swelling and inflammatory cell infiltration (*p* < 0.05) in mouse models of acute gout [45]. Corn silk (*Stigma madis*) is a style of maize (*Zea mays* L.) in the grass family [13] and causes diuresis, dispelling dampness and alleviating swelling. “Southern Yunnan Materia Medica” records its treatment of swelling and pain [46]. Modern research shows that corn silk contains sugars, flavonoids, mineral elements, volatile oils, alkaloids, amino acids, and other chemical components that have anti-oxidant [47], antibacterial [48], antitumor [49], blood sugar-lowering [50], and other pharmacological effects. A recent study found that corn silk can reduce serum uric acid levels by 26.69% (*p* < 0.05) and serum xanthine oxidase (XO) activity by 11.29% (*p* < 0.05) [51]. In addition, a study found that corn silk extract suppressed plasma uric acid in high salt-fed rats (*p* < 0.05) [13]. In China, corn production is abundant. Corn is a medicinal crop with a wide range of sources, is low-cost, and is easy to harvest. In daily life, corn silk is processed and used in beverages, oral liquids, and tea to relieve gout and other diseases, and is often used in combination with other medicines. For gout patients, the long-term use of traditional western medicine can be costly and have significant side effects. Turmeric and corn silk can make up for this deficiency as they are economical and easy to obtain, have few side effects, and can be taken for a long time. In addition, these compounds prevent gouty arthritis by delaying the onset and progression of the disease course. Therefore, in some circumstances, TCM has broad development prospects in supplementing and replacing western medicine.

Traditional culture has endured in China for thousands of years, and TCM has been integral to the country's traditions. Theories and thousands of years of practice, be it the same disease with different treatments, the theory of medicine and food homology, or other traditional Chinese medicine theories, have significantly protected the health of the country's people. Although modern medicine is mainstream in today's society, health problems persist. TCM has the potential to lead to breakthroughs in the health field, as evidenced by Youyou Tu, who invented artemisinin as a cure for malaria. We hope that TCM can better benefit people across the world.

## 5. Conclusion

Through network pharmacology and molecular docking, bisacumol, campesterol, and stigmasterol have been found to be essential turmeric compounds for gout treatment. These active ingredients may target protein processing in the endoplasmic reticulum through HSPA1B, HSP90AB1, and STUB1 proteins and play a significant role in treating gout. The essential compound of corn silk is mandenol, which may target the hippo signaling pathway to treat gout through CTNNB1, YWHAG, and YWHAZ proteins. Turmeric is a blood-activating medicine, and corn silk is a diuretic medicine. These compounds have different effects and applications but can treat the same disease through different pathways and targets. Hence, the scientific definition of the TCM theory is “same disease with different treatments.”

## Figures and Tables

**Table 1:** Database and software used in this study.

No.	Database and software	Function	Website
1	Traditional Chinese medicine systems pharmacology (TCMSP)	Screening herbal compounds	https://tcmspw.com/tcmspsearch.php
2	SEA database	Screening herbal compounds	https://sea.Bkslab.org/
3	Drugbank	Collection of disease targets	https://www.drugbank.ca/
4	DisGeNET	Collection of disease targets	http://www.disgenet.org/home/
5	GeneCards	Collection of disease targets	https://www.genecards.org/
6	OMIM	Collection of disease targets	https://www.omim.org/
7	PharmGkb	Collection of disease targets	https://www.pharmgkb.org/
8	R Packages	Visualized analysis	https://www.r-project.org/
9	Metascape	GO and KEGG analysis	https://metascape.org/
10	Cytoscape	Network construction	https://www.cytoscape.org

**Table 2:** Corresponding table of turmeric compounds and targets.

No.	Mol ID	MOl name	OB (%)	DL	Targets
1	MOL000449	Stigmasterol^*∗*^	43.83	0.76	IGHG1, RXRA, ADRA2A, SLC6A2, ADRB2, CHRM3, CHRM1, ADRB1, SCN5A, COLQ, CDC25A, GLRA1
2	MOL000493	Campesterol^*∗*^	37.58	0.71	CYP17A1, NPC1L1, RORC, CYP19A1, RORA
3	MOL000953	CLR	37.87	0.68	NA
4	MOL000955	Turmeronol A	59.42	0.08	CHRM3, CHRM1, COLQ, CHRM2, ADRA1B, CHRNA2, DRD5, APH1B, MAPK8, MAPK10, NR1D1
5	MOL000966	Turmeronol B	35.84	0.08	DRD1, APH1B, DRD4, EPHX1, DRD5
6	MOL000963	Bisacumol^*∗*^	31.41	0.07	AR, RXRA, COLQ, APH1B, CYP17A1

Note. “^*∗*^” indicates important compounds.

**Table 3:** Corresponding table of corn silk compounds and targets.

No.	Mol ID	MOl name	OB (%)	DL	Targets
1	MOL010862	*α*-Tocopherylquinone	35.91	0.50	INSRR, DAGLA, CALCA, MTTP, IL6R, GPBAR1, CYP19A1, ATP6AP2, NPC1L1, MAPK8, MAPK9, BCL2, DRD2, DRD3, PGA4, KCNA5, SEMA4D
2	MOL013356	Stigmasta-4,22-diene-3beta,6beta-diol	39.32	0.79	NPC1L1, RORC, CYP19A1, RORA, CHRM2, CYP17A1, COLQ, KCNA3, DRD2, KCNA5, MDM4, CYP24A1, MAPK8, CHRM4, CHRM1, CHRM3, CDK8, EPHX1
3	MOL013357	(3S,6R,8S,9S,10R,13R,14S,17R)-17-[(1R,4R)-4-ethyl-1,5-dimethylhexyl]-10,13-dimethyl-2,3,6,7,8,9,11,12,14,15,16,17-dodecahydro-1H-cyclopenta[a]phenanthrene-3,6-diol	34.37	0.78	NA
4	MOL013359	Stigmasta-7-en-3-ol	37.42	0.75	NA
5	MOL001494	Mandenol^*∗*^	42.00	0.19	CYP17A1, CYP19A1, APH1B, FFAR1, FABP1, GRIN2B, SCD, PTGIR, KIF11, SLC6A15, CETP, BRD4, BRD2, BRD3, PICK1, TRAF5
6	MOL001749	ZINC03860434	43.59	0.35	SCN5A, CHRM3, ADRB2, CHRM1
7	MOL003044	Chrysoeriol	35.85	0.27	AR, CDK2, CAMTA2, SERPINB2, ABCB1, ABCG2, TTR, COLQ, CYP19A1, MCL1, DRD4, GPR35, CNOT1, CABLES1, EID3
8	MOL003059	Kryptoxanthin	47.25	0.57	CDC25A, GLRA1, CYP19A1, RORC, NPC1L1, CYP17A1
9	MOL000359	Sitosterol	36.91	0.75	NPC1L1, CYP17A1, RORC, CYP19A1, RORA, CHRM2, COLQ, CDC25A, GLRA1, CDC25B, DRD2
10	MOL000449	Stigmasterol	43.83	0.76	IGHG1, RXRA, ADRA2A, SLC6A2, ADRB2, CHRM3, CHRM1, ADRB1, SCN5A, ADRA1A, CHRM2, ADRA1B, CHRNA7, NPC1L1, CYP17A1, CYP19A1, RORC, RORA, COLQ, CDC25A, GLRA1
11	MOL000006	Luteolin	36.16	0.25	Ar, RELA, CCND1, CDKN1A, IL10RB, RB1, CDK4, TNF, JUN, IL6R, TP53, NFKBIA, APP, MCL1, BIRC5, IL2RA, CCNB1, IL4R, XIAP, SLC2A4, INSRR, TTR, ABCG2, COLQ, ABCB1, CYP19A1, GPR35, DRD4, SERPINB2, KDM4E, CNOT1, GSKIP
12	MOL006756	Schottenol	37.42	0.75	NPC1L1, RORC, CYP19A1, CYP17A1, COLQ, CHRM2, RORA, NR1I3, GLRA1, CDC25A, PREPL, CDC25B, ATP12A, FABP1

*Note*. “^*∗*^” indicates important compounds.

**Table 4:** Targets of turmeric for gout.

Number	Hub name	Degree
1	HSPA8^*∗*^	102
2	VCP	94
3	HSP90AB1^*∗*^	92
4	HSPA5^*∗*^	82
5	CUL1	74
6	SP1	71
7	PRKDC	71
8	ACTB	70
9	HNRNPK	68
10	ABL1	68
11	HNRNPU	67
12	HSPA1B^*∗*^	65
13	HSPA1A	65
14	NFKB1	64
15	DDX5	62
16	GAPDH	62
17	CDKN1A	61
18	HSPA9	61
19	PPP1CA	61
20	PARP1	60
21	VHL	59
22	STUB1^*∗*^	58
23	RPS27A	57
24	HSPD1	56
25	CUL2	56
26	ARRB1	55
27	XRCC6	52
28	CDK1	52
29	SIRT1	51
30	HIF1A	51
31	RAF1	51
32	FLNA	49

*Note*. “^*∗*^” indicates important targets.

**Table 5:** Targets of corn silk for gout.

Number	Hub name	Degree
1	YWHAZ^*∗*^	430
2	NPM1	409
3	HNRNPA1	334
4	HSPA8	278
5	CTNNB1^*∗*^	271
6	YWHAG^*∗*^	268
7	RPS27A	238
8	HSPA4	225
9	TUBB	200
10	GAPDH	173

*Note*. “^*∗*^” indicates important targets.

**Table 6:** Molecular docking scoring.

Target protein	Compound			
	Bisacumol^*∗*^	Campesterol^*∗*^	Stigmasterol^*∗*^	Mandenol^#^
HSPA1B^*∗*^	8.60	8.97	7.61	——
HSP90AB1^*∗*^	6.75	5.18	5.35	——
STUB1^*∗*^	6.93	6.83	5.64	——
YWHAG^#^	——	——	——	7.56
CTNNB1^#^	——	——	——	6.14
YWHAZ^#^	——	——	——	6.04

*Note.* “^*∗*^” represents turmeric. “^#^” represents corn silk.

## Data Availability

The data used to support the findings of this study are included within the article and in the supplementary information.

## Supplementary Materials

Supplementary Materials Fig.S1: research schematic. Fig.S2: Venn diagram of disease targets and compound targets. (A) Venn diagram of intersection targets of 6 6 gout disease databases. (B) Venn diagram of the intersection targets of the turmeric compound targets and the gout targets. (C) Venn diagram of the intersection targets of the corn silk compound targets and the gout targets. Fig.S3: “medicine-compounds -target-disease” network. (A) Turmeric network diagram. (B) corn silk network diagram. Fig.S4: the PPI network of turmeric -gout targets. Note: degree centrality (DC), closeness centrality (CC), betweenness centrality (BC), network centrality (NC), and local average connectivity (LAC). Fig.S5: the PPI network of corn silk -gout targets. Note: degree centrality (DC), closeness centrality (CC), betweenness centrality (BC), network centrality (NC), and local average connectivity (LAC).
