# Supplementary material for: Treatment of Gout with TCM Using Turmeric and Corn Silk: A Concise Review Article and Pharmacology Network Analysis
Source: Evid Based Complement Alternat Med. 2022 Oct 14;2022:3143733. doi: 10.1155/2022/3143733 (PMC9586733; doi:10.1155/2022/3143733)
Supplement: Supplementary Materials — Fig.S1: research schematic. Fig.S2: Venn diagram of disease targets and compound targets. (A) Venn diagram of intersection targets of 6 6 gout disease databases. (B) Venn diagram of the intersection targets of the turmeric compound targets and the gout targets. (C) Venn diagram of the intersection targets of the corn silk compound targets and the gout targets. Fig.S3: “medicine-compounds -target-disease” network. (A) Turmeric network diagram. (B) corn silk network diagram. Fig.S4: the PPI network of turmeric -gout targets. Note: degree centrality (DC), closeness centrality (CC), betweenness centrality (BC), network centrality (NC), and local average connectivity (LAC). Fig.S5: the PPI network of corn silk -gout targets. Note: degree centrality (DC), closeness centrality (CC), betweenness centrality (BC), network centrality (NC), and local average connectivity (LAC). [file 3143733.f1.doc]

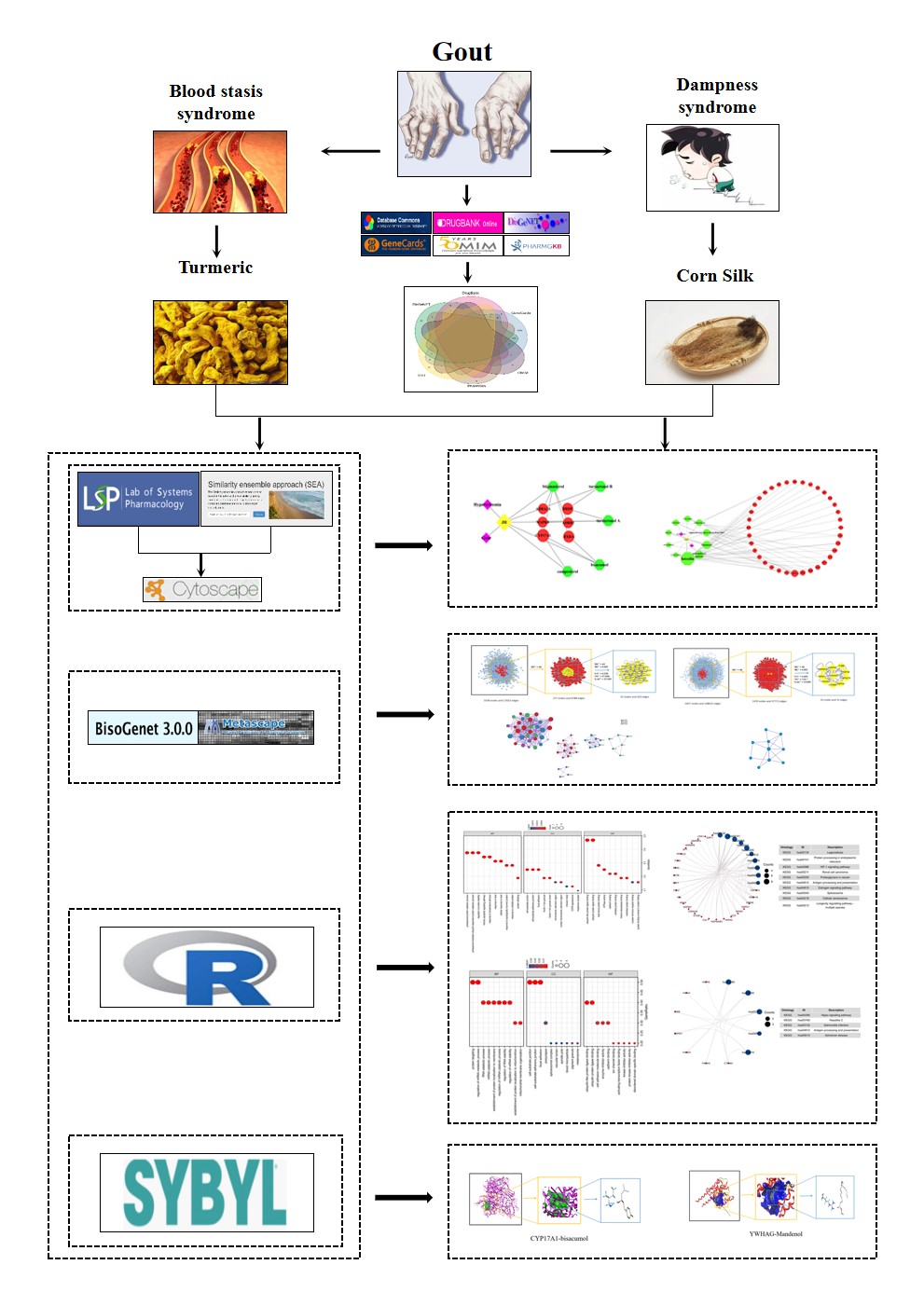


**Fig.S1**: Research schematic.


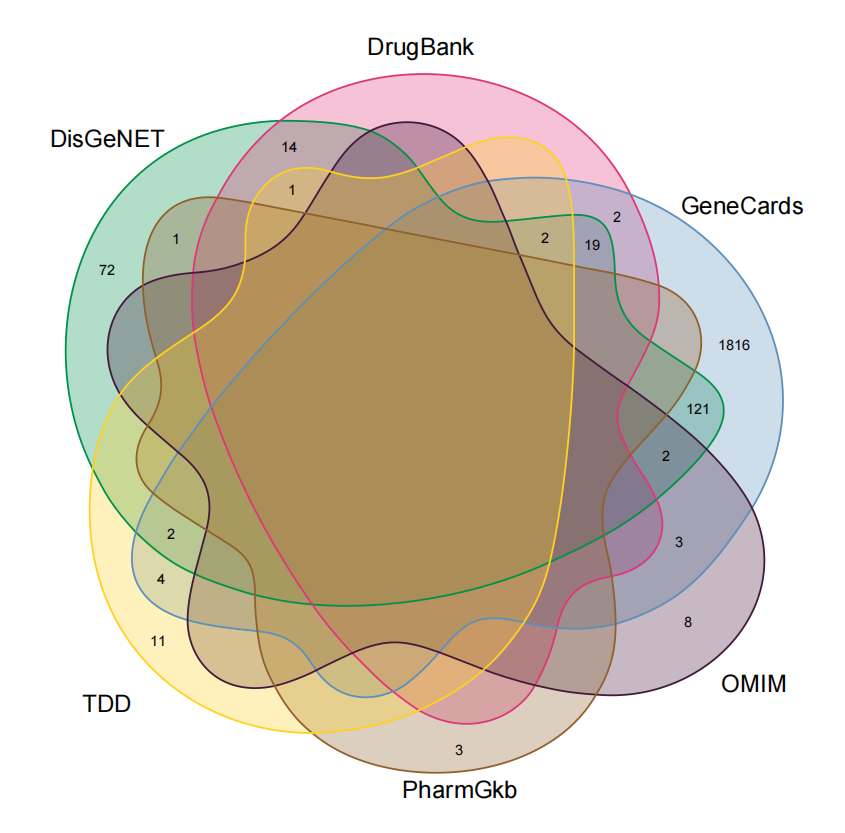

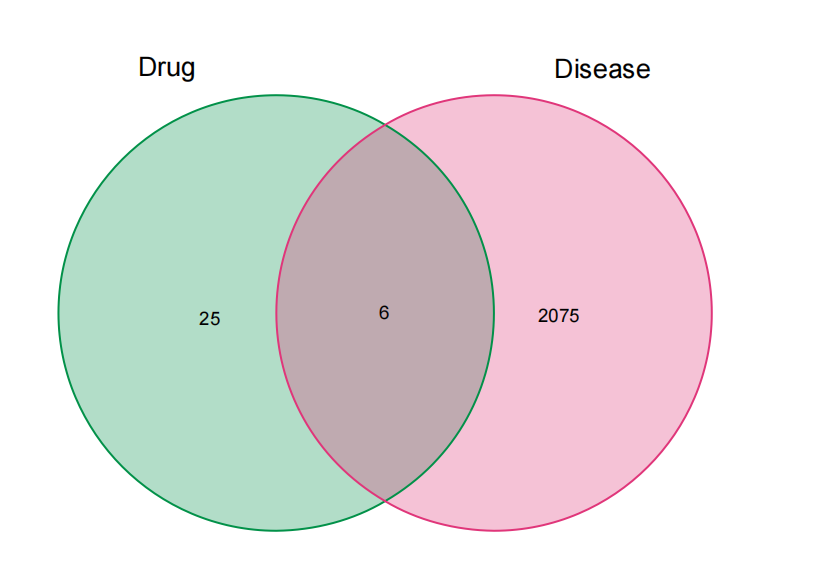

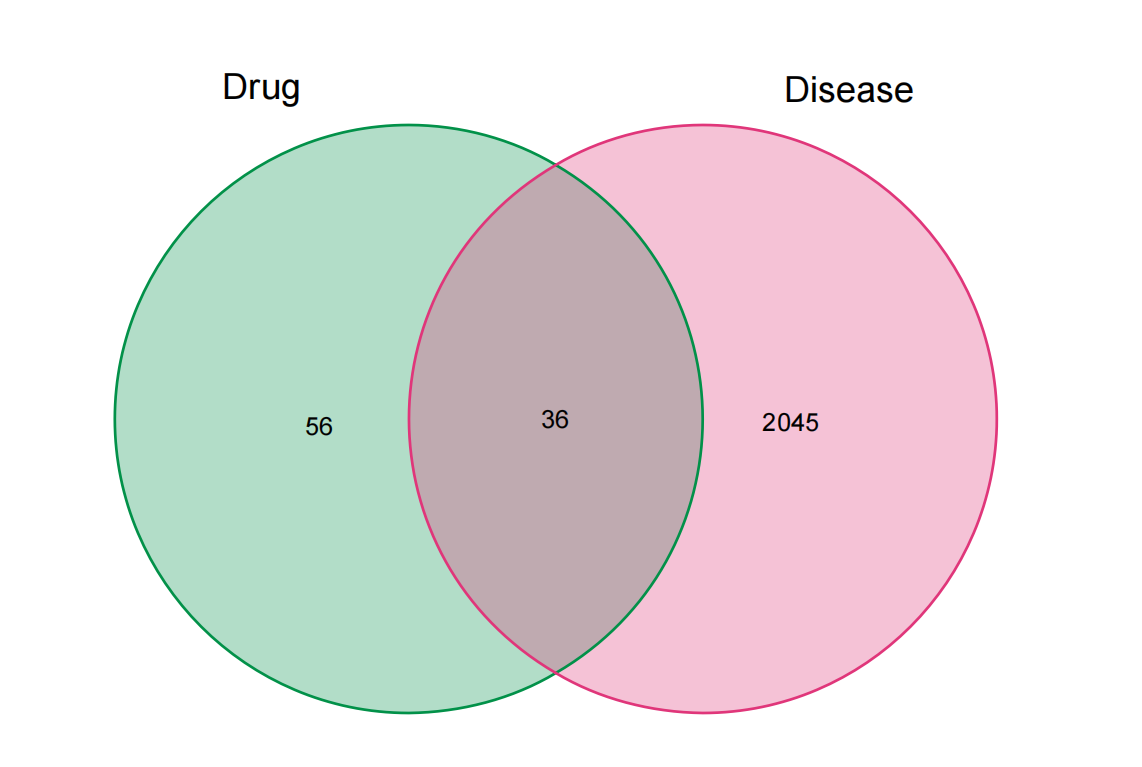


(A) (B) (C)

**Fig.S2**: Venn diagram of disease targets and compound targets. (A)Venn diagram of intersection targets of 6 6 gout disease databases. (B)Venn diagram of the intersection targets of the turmeric compound targets and the gout targets. (C)Venn diagram of the intersection targets of the corn silk compound targets and the gout targets.


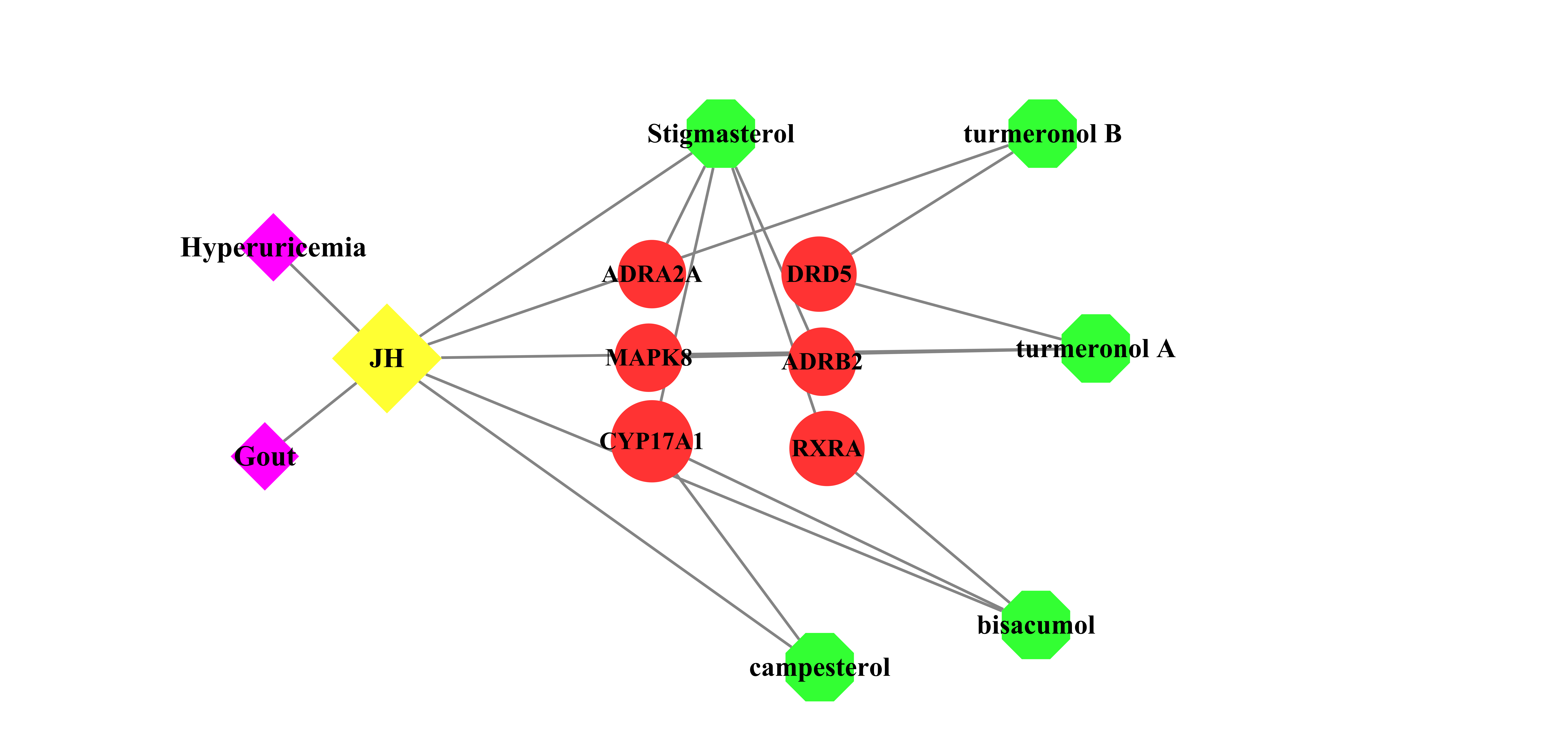

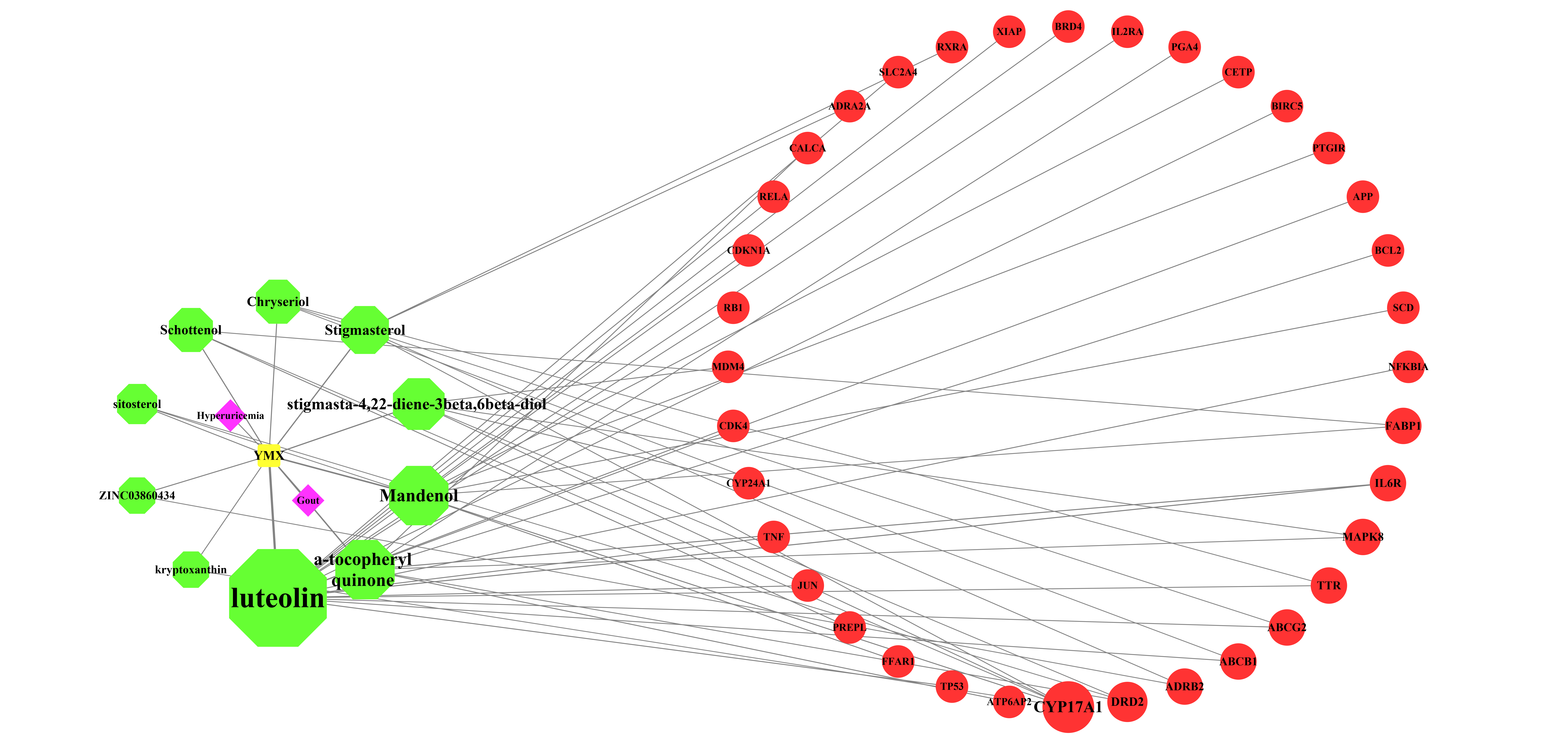
（A） （B）

**Fig.S3**: “Medicine-compounds -target-disease" network. （A）Turmeric network diagram. (B) corn silk network diagram.


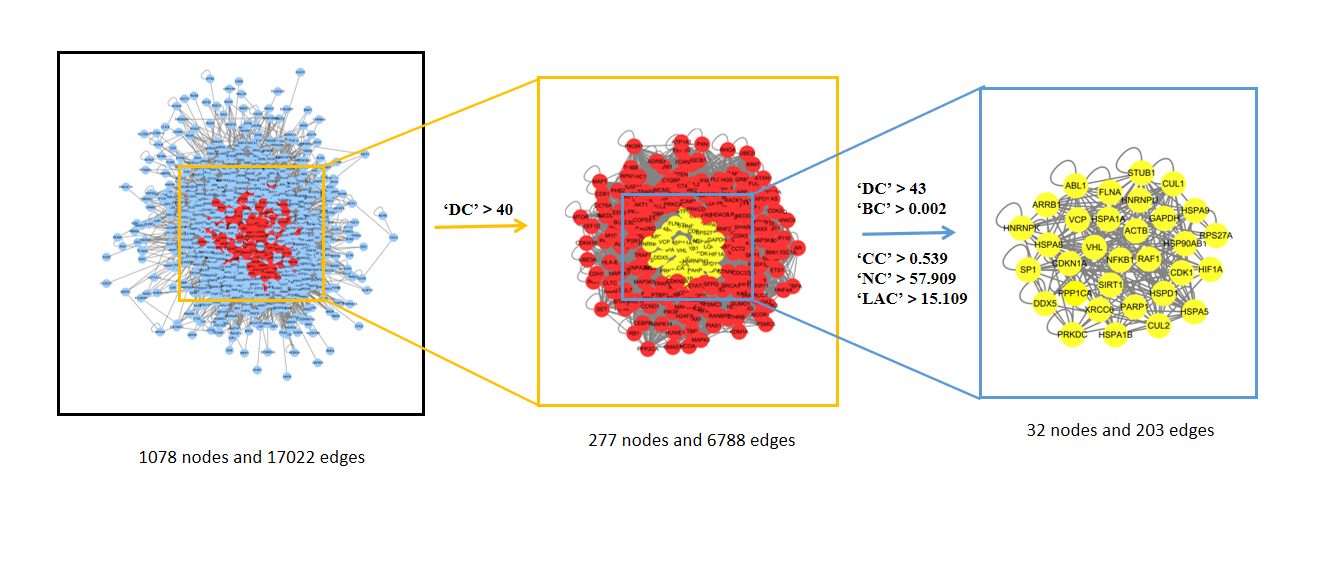


**Fig.S4**: The PPI network of turmeric -gout targets. Note: degree centrality (DC), closeness centrality (CC), betweenness centrality (BC), network centrality (NC) and Local average connectivity (LAC).


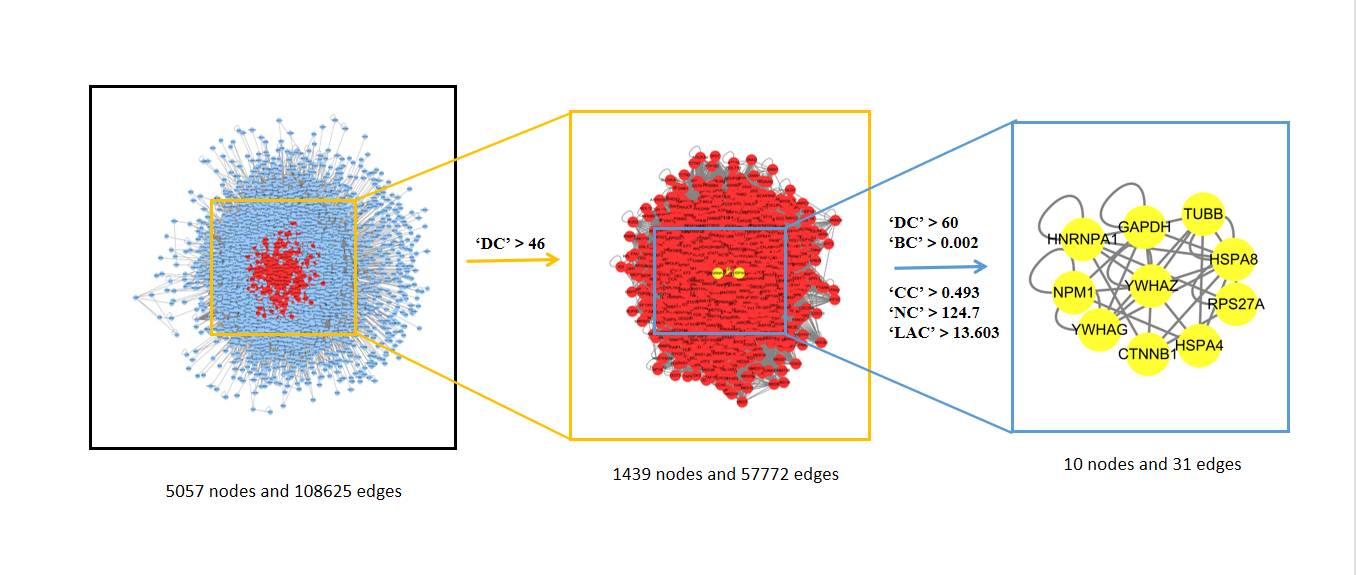


**Fig.S5**: The PPI network of corn silk -gout targets. Note: degree centrality (DC), closeness centrality (CC), betweenness centrality (BC), network centrality (NC) and Local average connectivity (LAC).
